# Gate-Localized Fluorination Enables Enhancement-Mode AlGaN/GaN High-Electron Mobility Transistors

**DOI:** 10.1021/acsaelm.5c02507

**Published:** 2026-01-24

**Authors:** Do Wan Kim, Byungsoo Kim, Yongjoo Cho, Seokho Kim, Yao Gong, Yongmin Baek, Byungjoon Bae, Young-Kyun Noh, Seongwan Bae, Dong Hyuk Park, Kyusang Lee

**Affiliations:** † Department of Electrical and Computer Engineering, 2358 University of Virginia, Charlottesville, Virginia 22904, United States; ‡ Future Semiconductor Business Inc., Charlottesville, Virginia 22902, United States; § IVWorks Co., Ltd, Daejeon 34122, South Korea; ∥ Department of Chemical Engineering, 26718 Inha University, Incheon 22212, Republic of Korea; ⊥ Program in Biomedical Science & Engineering, Inha University, Incheon 22212, Republic of Korea; # Department of Materials Science and Engineering, University of Virginia, Charlottesville, Virginia 22904, United States

**Keywords:** AlGaN/GaN HEMTs, enhancement-mode operation, CHF_3_ plasma treatment, threshold voltage engineering, gate recess

## Abstract

Gallium nitride (GaN)-based high-electron-mobility transistors (HEMTs) are key to high-power and high-frequency electronics owing to their wide bandgap, high breakdown field, and ability to form a high-density two-dimensional electron gas (2DEG) at the AlGaN/GaN interface. For power-switching systems, enhancement-mode (E-mode) operation, where devices remain normally off at zero gate bias, is preferred for intrinsic failsafe behavior and reduced standby power. However, conventional E-mode strategies, such as deep gate recessing or p-type gate insertion, often introduce fabrication complexity, surface damage, and long-term instability. Here, we demonstrate a gate-localized CHF_3_ plasma process that simultaneously produces a self-limiting recess with a fluorine-terminated surface, enabling a normally off AlGaN/GaN HEMT. Fluorine incorporation compensates polarization-induced charges and drives a positive shift in threshold voltage (*V*_th_), whereas hydrogen species generated during plasma exposure passivate etch-induced Ga-related defects and suppress interface-trap formation. By confining plasma exposure to the gate region, this method mitigates surface degradation and charge trapping typically observed with CF_4_ processing, achieving precise and stable *V*_th_ control without deep gate recessing. The fabricated devices exhibit normally off operation while maintaining low gate leakage under bias stress. This single step, lithographically confined approach offers a practical route toward E-mode GaN HEMTs for energy-efficient, high-frequency, and high-power electronic systems.

## Graphical abstract

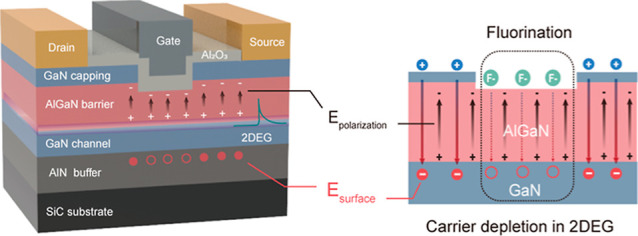

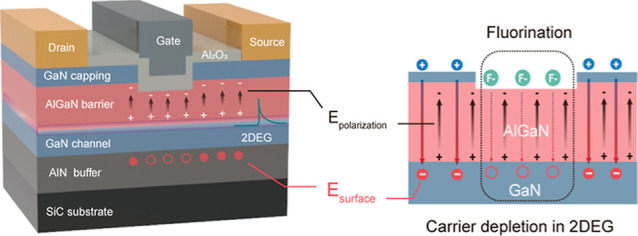

## Introduction

Gallium nitride (GaN)-based high electron mobility transistors (HEMTs) have emerged as essential components in high-voltage and high-frequency electronics due to their wide bandgap, high breakdown field, and the formation of a robust two-dimensional electron gas (2DEG) at the AlGaN/GaN heterointerface.^1–3^ These attributes enable devices with high power density, large current capability, and low on-resistance, making GaN HEMTs well-suited for radiofrequency (RF) amplification and power-switching systems.^4^ However, AlGaN/GaN HEMTs inherently operate in depletion-mode (D-mode) due to the polarization-induced 2DEG remaining populated even at zero gate bias.^5,6^ For power-electronic applications, such normally on behavior introduces fundamental safety and failsafe concerns, since current continues to flow during the off-state, potentially leading to catastrophic circuit damage if the gate driver fails.^7^ In addition, D-mode operation compromises energy-conversion efficiency because maintaining a negative gate bias requires additional power rails and incurs gate-drive losses during high-frequency switching.^8^ Consequently, enhancement-mode (E-mode) GaN HEMTs, which are normally off at zero gate voltage, have gained prominence for their improved safety, energy efficiency, and system-level compatibility.^9^

Several strategies have been explored to induce the E-mode operation. Gate recessing, achieved by partial etching of the AlGaN barrier beneath the gate, reliably shifts the threshold voltage (*V*_th_) in the positive direction;^10–13^ however, it requires nanometer-scale etch precision and often introduces plasma-induced defects that degrade surface morphology and long-term reliability.^14–16^ Alternatively, incorporating a p-type GaN gate layer achieves channel depletion via band structure modification;^17^ nevertheless, this approach necessitates complex epitaxial growth and selective etching processes and remains susceptible to gate leakage and threshold drift under prolonged bias stress.

To mitigate these fabrication complexities, fluorine-based plasma treatments have emerged as a simpler, postgrowth strategy for threshold modulation.^18–20^ In this process, negatively charged fluorine atoms incorporate near the AlGaN surface, partially compensating for polarization-induced charges and thereby depleting the underlying 2DEG. CF_4_-based plasmas, in particular, have been widely explored for this purpose. However, it often leads to surface damage, charge trapping, elevated gate leakage, and threshold voltage instability, issues that compromise reproducibility and reliability.

Here, we introduce a gate-localized CHF_3_ plasma treatment that overcomes these limitations by combining the controlled fluorine incorporation with mild surface chemistry. The CHF_3_ plasma simultaneously induces a self-limiting, nanometer-precision recess and terminates the AlGaN surface with fluorine, producing a stable positive shift in *V*_th_ without resorting to deep etching. Moreover, hydrogen species generated during the plasma process effectively passivate plasma-induced Ga-related vacancy defects by forming stable H–N complexes with nitrogen atoms adjacent to the vacancy sites, thereby improving device reliability.^21–24^ By spatially confining plasma exposure to the gate region, this process reduces parasitic surface modification elsewhere on the device, enabling precise and reproducible control of the threshold voltage. The gate-confined fluorination strategy thus provides a robust route toward E-mode AlGaN/GaN HEMTs, combining the simplicity of plasma processing with the precision of self-limiting chemistry.

## Experimental Section

Figure 1a presents the schematic architecture of the fabricated AlGaN/GaN HEMT, which incorporates a gate-localized CHF_3_ plasma treatment precisely confined to the gate region. The AlGaN/GaN heterostructure was epitaxially grown on a SiC substrate by molecular beam epitaxy (MBE), comprising a 400 nm AlN buffer layer, a 150 nm unintentionally doped GaN channel, an 18 nm Al_0.3_Ga_0.7_N barrier, and a 3 nm GaN cap. The device adopts a 10-finger interdigitated configuration with a gate length of 1000 μm and a width of 15 μm.

**Figure 1:**
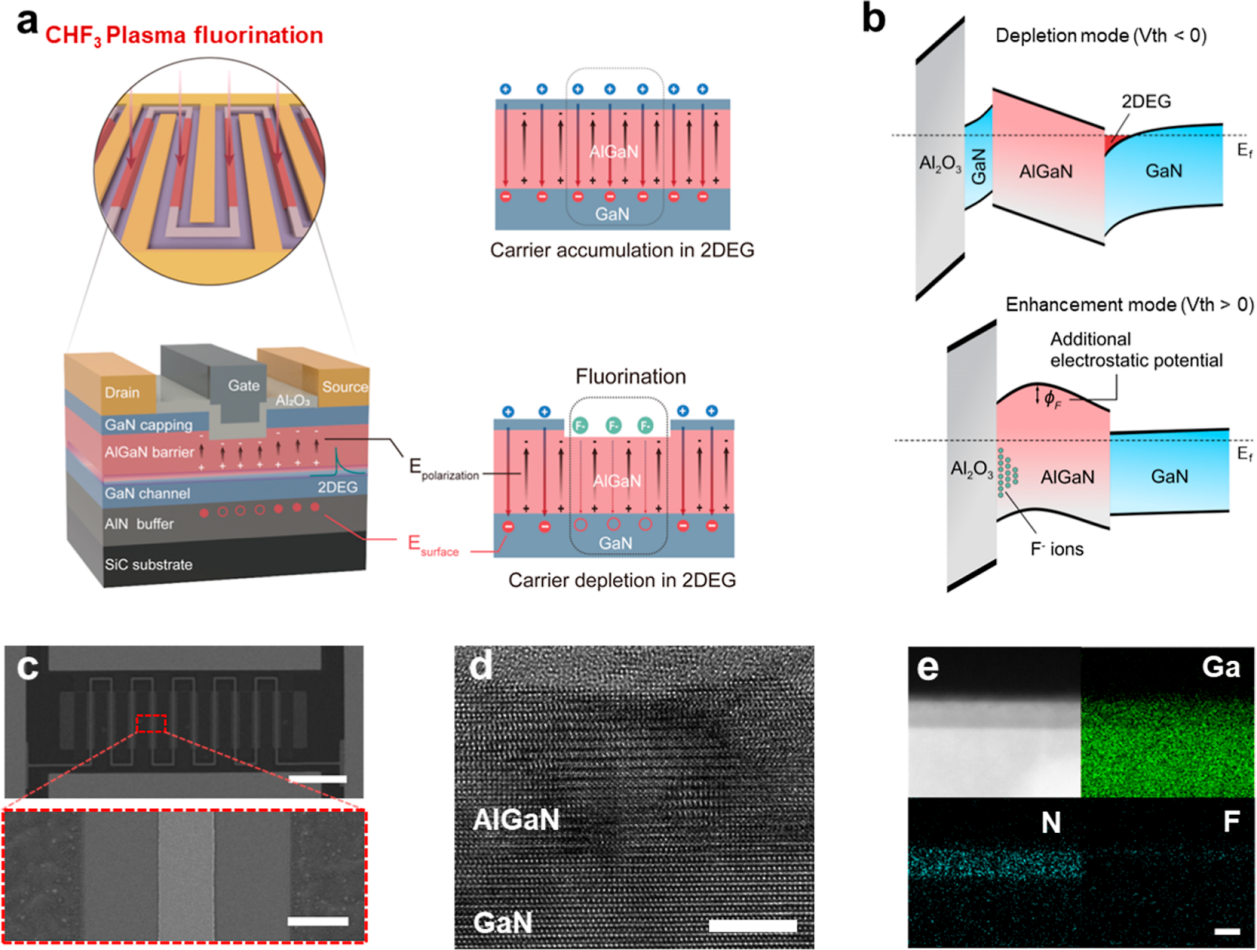
Structural and compositional characterization of a CHF_3_-plasma-treated AlGaN/GaN HEMT. (a) Schematic of the fabricated 10-finger AlGaN/GaN HEMT in which selective-area CHF_3_ plasma treatment is confined to the gate region. The localized exposure introduces fluorine and induces a self-limited nanometer-scale recess, modifying the electrostatics at the AlGaN/GaN interface, suppressing the polarization-induced 2DEG, and thereby enables normally off operation. (b) Energy band diagrams of the untreated (top) and CHF_3_-treated (bottom) devices, showing how fluorine incorporation on the AlGaN barrier modifies the band profile. (c) SEM images of the fabricated 10-finger AlGaN/GaN HEMT device following selective-area CHF_3_ plasma treatment (top). The gate region is clearly defined between the source and drain fingers, with the treated area confined beneath the gate (bottom). Scale bars: 100 μm (top), 4 μm (bottom). (d) Cross-sectional TEM image of the gate region of the CHF_3_-treated HEMT at 100 W, confirming the removal of the GaN capping layer and a partial thinning of the AlGaN barrier, leaving ∼10 nm of AlGaN above the GaN channel. (e) STEM image and corresponding EDS elemental mapping (Ga, Al, and F), indicating fluorine predominantly localized near the AlGaN surface beneath the gate.

The fabrication process for the AlGaN/GaN HEMT began with device isolation, which was achieved through mesa formation by using Cl_2_/BCl_3_ dry etching in an inductively coupled plasma reactive ion etching (ICP–RIE) system. Ohmic contacts for the source and drain were deposited by e-beam evaporation of Ti/Al/Ni/Au (25/140/80/50 nm), followed by rapid thermal annealing (RTA) at 780 °C for 32 s in a nitrogen atmosphere to achieve low-resistance contacts. The exposed AlGaN surface in the gate region was then subjected to a selective-area CHF_3_ plasma treatment under three distinct conditions, each conducted for 600 s at 20 °C with a chamber pressure of 20 mTorr and a CHF_3_ gas flow rate of 60 sccm. The first condition employed an RF power of 30 W, while the second increased the RF power to 100 W. For comparison, a recessed reference device with an equivalent etch depth of ∼10 nm, comparable to that obtained under the 100 W condition, was fabricated using an ICP power of 300 W and RF power of 5 W under a Cl_2_/BCl_3_ gas mixture (18/2 sccm) at 10 mTorr and 20 °C. Following surface modification, a 10 nm Al_2_O_3_ passivation layer was deposited by atomic layer deposition (ALD) and annealed at 300 °C for 1 h in ambient conditions. The metal–insulator–semiconductor (MIS) gate was then formed by deposition of a 40 nm Ni layer, followed by Ni/Au (20/50 nm) pads defining the source, drain, and gate electrodes.

## Results and Discussion

The band diagrams for the D- and E-mode AlGaN/GaN HEMTs are illustrated in Figure 1b. In the D-mode operation (top panel) without the recess or plasma treatment process, the polarization-induced positive charges at the AlGaN/GaN interface induce a 2DEG channel. Following CHF_3_ plasma treatment (bottom panel), fluorine ions (F^–^) incorporated near the AlGaN surface form Al–F_*x*_ bonds that introduce immobile negative charges.^25,26^ These charges effectively compensate the built-in positive polarization field, flattening the band profile of the AlGaN/GaN 2DEG channel by shifting the conduction band above the Fermi level at zero gate bias, thereby depleting the carriers. As a result, the *V*_th_ shifts in the positive direction, enabling normally off operation.

A scanning electron microscopy (SEM) image of the fabricated multifinger configuration is shown in Figure 1c with a magnified view of the gate region (∼3 μm) located between the source and drain electrodes. Cross-sectional transmission electron microscopy (TEM) of the gate region (Figure 1d) revealed a well-defined AlGaN/GaN heterostructure, confirming that the interfacial structure remains intact following CHF_3_ plasma treatment. The GaN capping and AlGaN barrier layers exhibit a controlled partial etch, leaving approximately 10 nm-thick AlGaN beneath the gate. The corresponding scanning transmission electron microscopy (STEM) image and energy-dispersive X-ray spectroscopy (EDS) elemental maps (Figure 1e) further confirm the spatial distribution of fluorine, aluminum, and gallium. Fluorine is predominantly concentrated near the surface of the AlGaN layer with slight diffusion into the underlying GaN channel. These structural and compositional analyses indicate that the CHF_3_ plasma treatment achieves targeted fluorine incorporation and polarization charge modulation while preserving the structural integrity of the AlGaN/GaN heterointerface.

Atomic force microscopy (AFM) was performed to evaluate the surface morphology of AlGaN/GaN heterostructures following CHF_3_ plasma treatment under varying conditions (Figure 2). The untreated reference sample (Figure 2a) exhibited an atomically smooth surface with a root-mean-square (RMS) roughness of 0.57 nm. Treatment with an RF power of 30 W exhibited a slightly reduced RMS roughness of 0.35 nm and produced a shallow etch of the GaN capping layer of approximately 2.5 nm (Figure 2b). At a higher RF power of 100 W, the 3 nm GaN capping layer was completely removed, and the underlying AlGaN barrier was thinned by approximately 8.0 nm, with an RMS roughness of 0.58 nm (Figure 2c). Line profiles averaged over five horizontal cross sections of the AFM images (Figure 2d) confirm the progressive increase in recess depth from ∼2.5 nm at 30 W to ∼11.0 nm at 100 W. These results indicate that CHF_3_ plasma treatment not only incorporates fluorine into the AlGaN layer but also enables a controlled surface recess while preserving smoothness and minimizing plasma-induced damage.

**Figure 2:**
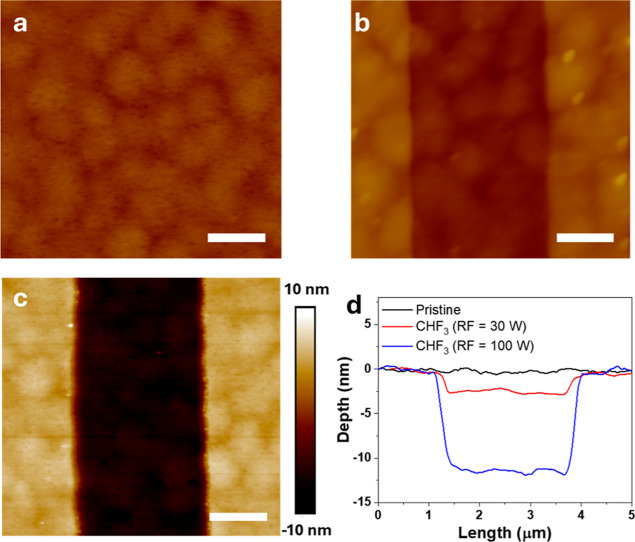
Surface morphologies of the CHF_3_-plasma-treated AlGaN surface characterized by AFM. (a) Untreated reference sample showing a smooth surface with a root-mean-square (RMS) roughness 0.57 nm. (b) RF plasma exposure at 30 W induces a shallow ∼2.5 nm recess associated with minor etching of the GaN capping layer with RMS roughness reduced to 0.35 nm. (c) RF plasma treatment at 100 W produces a ∼11.0 nm recess, indicating complete removal of the 3 nm GaN capping layer and additional thinning (∼8.0 nm) of the AlGaN barrier with a RMS roughness of 0.58 nm (scale bar: 1 μm). (d) Averaged line profiles extracted from five evenly spaced horizontal cross sections in the AFM images, confirming the progressive increase in recess depth with RF power.

To elucidate the chemical mechanism underlying threshold-voltage modulation, X-ray photoelectron spectroscopy (XPS) analysis was performed on AlGaN/GaN HEMTs subjected to CHF_3_ plasma treatment at RF powers of 30 and 100 W. The F 1s spectra (Figure 3a) exhibit a distinct peak at ∼684.7 eV corresponding to the metal–F bonding, where stronger RF power results in a higher degree of fluorine incorporation and enhanced metal–F bond formation.^27,28^ In contrast, the pristine sample exhibited no detectable F 1s signal, confirming the absence of fluorine prior to plasma exposure. The Al 2p spectra (Figure 3b,c) show negligible changes in the Al–N bond intensity and peak position while the Al–F_*x*_ component (74.3–74.7 eV) becomes more pronounced, with its relative intensity increasing from 31.4% at 30 W to 41.5% at 100 W, indicating stronger fluorination at elevated plasma power.^29^

**Figure 3:**
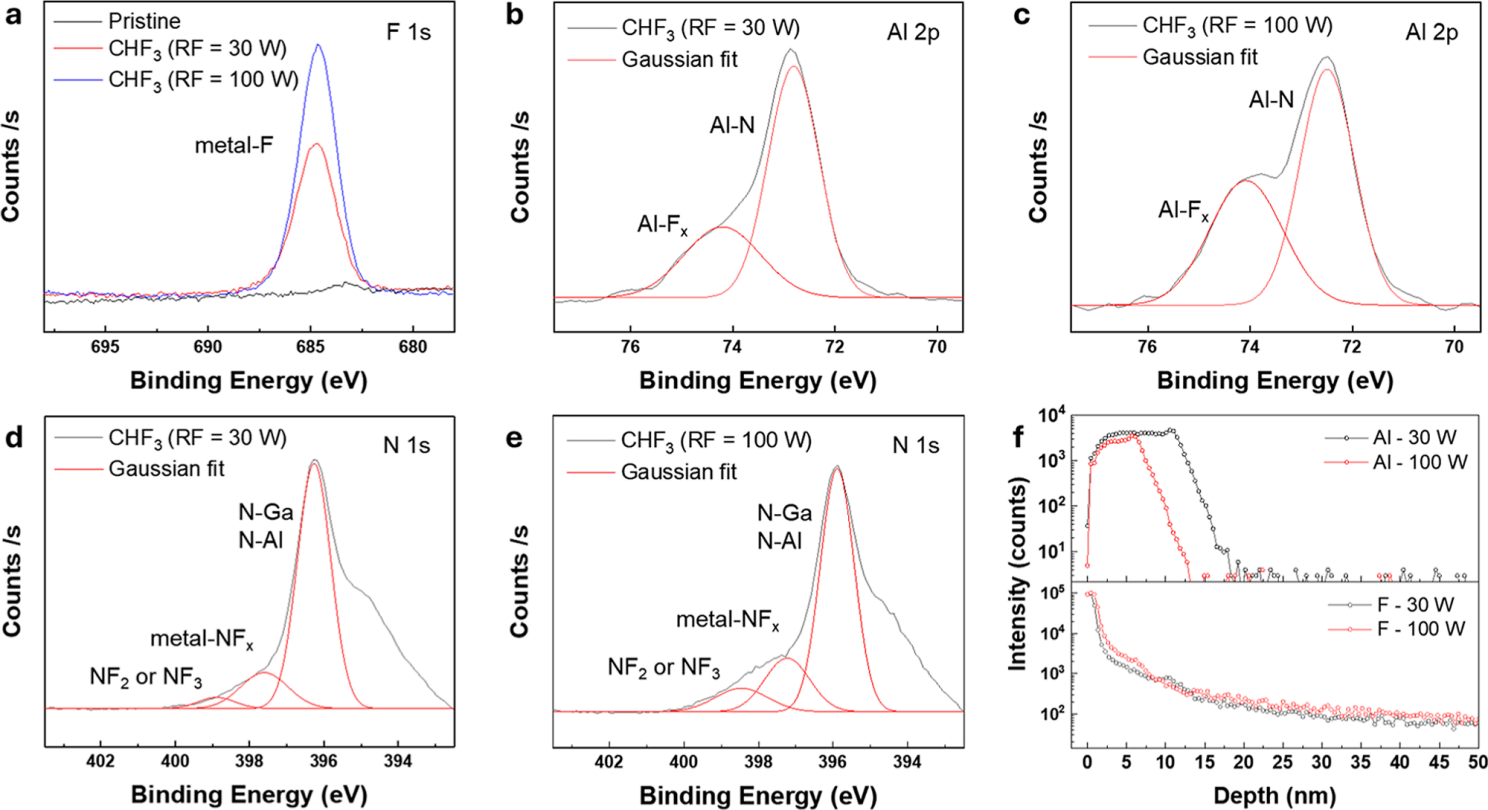
XPS and SIMS analysis of fluorination induced by CHF_3_ plasma treatment. (a) F 1s spectra showing the metal–F bond at ∼684.7 eV with increased intensity at higher RF power (100 W). (b,c) Al 2p spectra for samples treated at 30 W (b) and 100 W (c). The Al–N bond (∼72.5–72.8 eV) shows a minimal change in intensity, while the Al–F_*x*_ component (74.3–74.7 eV) increases from 31.4% to 41.5% with RF power. (d) Ga 2p_3/2_ XPS spectra showing a ∼0.45 eV binding-energy shift when the RF power increases from 30 to 100 W, indicating stronger fluorine incorporation. (e) N 1s spectra for the 100 W treatment, overlap with the Ga Auger peak (394.9), resolved into –N–Al (396.0 eV), –N–Ga (397.5 eV), and –NH (399.0 eV) states. Quantitative fitting shows that the –NH component increases to 4.19% at an RF power of 100 W. (f) SIMS depth profiles of Al and fluorine in AlGaN/GaN HEMTs after CHF_3_ plasma treatment. The fluorine penetration depth is ∼7 nm in both samples.

During the fluorine-based RIE process, surface etching generates defects such as Ga vacancies,^30^ which act as acceptor-like surface states and consequently drive the Fermi level of GaN toward the valence band.^31,32^ This characteristic is evidenced by the Ga 2p_3/2_ core-level XPS spectra, which exhibit a positive binding-energy shift of approximately 0.45 eV (Figure 3d).^33,34^ The N 1s core-level spectra overlap with the Ga LMM Auger peak (394.9 eV) and can be decomposed into contributions from –N–Al (396.0 eV), –N–Ga bonds (397.5 eV), and –NH (399.0 eV) components (Figure 3e).^35–38^ Quantitative analysis shows that the –NH fraction increases from 1.29% to 4.19% as the RF power is raised from 30 to 100 W, indicating enhanced hydrogen incorporation at near-surface sites. This hydrogen passivation at near-surface defect sites suppresses the electronic activity of residual trap states, resulting in a more uniform and less perturbed GaN surface potential.^21,25^

Depth profiling by secondary ion mass spectrometry (SIMS) analysis further clarifies the spatial distribution of fluorine within the heterostructure (Figure 3f). The Al signal identifies effective AlGaN barrier thicknesses of 18.4 and 13.1 nm for 30 and 100 W plasma treatment, respectively, consistent with TEM and AFM measurements. The reduced thickness at higher power reflects enhanced ion-assisted etching of the AlGaN barrier. Regardless of this difference in the AlGaN thickness after plasma treatment, both samples exhibit a comparable fluorine diffusion depth of ∼7 nm. This consistency indicates that the apparent variation arises from plasma-induced thinning rather than from differential fluorine diffusion. These results demonstrate that fluorination is governed by a self-limiting chemical process rather than by the ion bombardment energy.

The electrical characteristics of the fabricated AlGaN/GaN HEMTs were evaluated through transfer, gate leakage, and output measurements, enabling systematic assessment of threshold-voltage modulation and the transition from depletion- to enhancement-mode conduction (Figure 4). Transfer and transconductance curves were obtained by sweeping the gate voltage from −9 to 2 V at a drain-source bias of 1.0 V. At room temperature, the untreated AlGaN/GaN HEMT exhibits a *V*_th_ of −5.70 V (Figure 4a), consistent with conventional D-mode operation. Following CHF_3_ plasma exposure at an RF power of 30 W, *V*_th_ shifts to −3.25 V (Figure 4c), corresponding to a positive shift of approximately 2.5 V. Increasing the RF power to 100 W produces a substantially larger shift, driving *V*_th_ to +2.00 V and thereby enabling E-mode operation (Figure 4d). No measurable degradation in the device characteristics was observed following prolonged ambient exposure or postfabrication thermal annealing at 300 °C.

**Figure 4:**
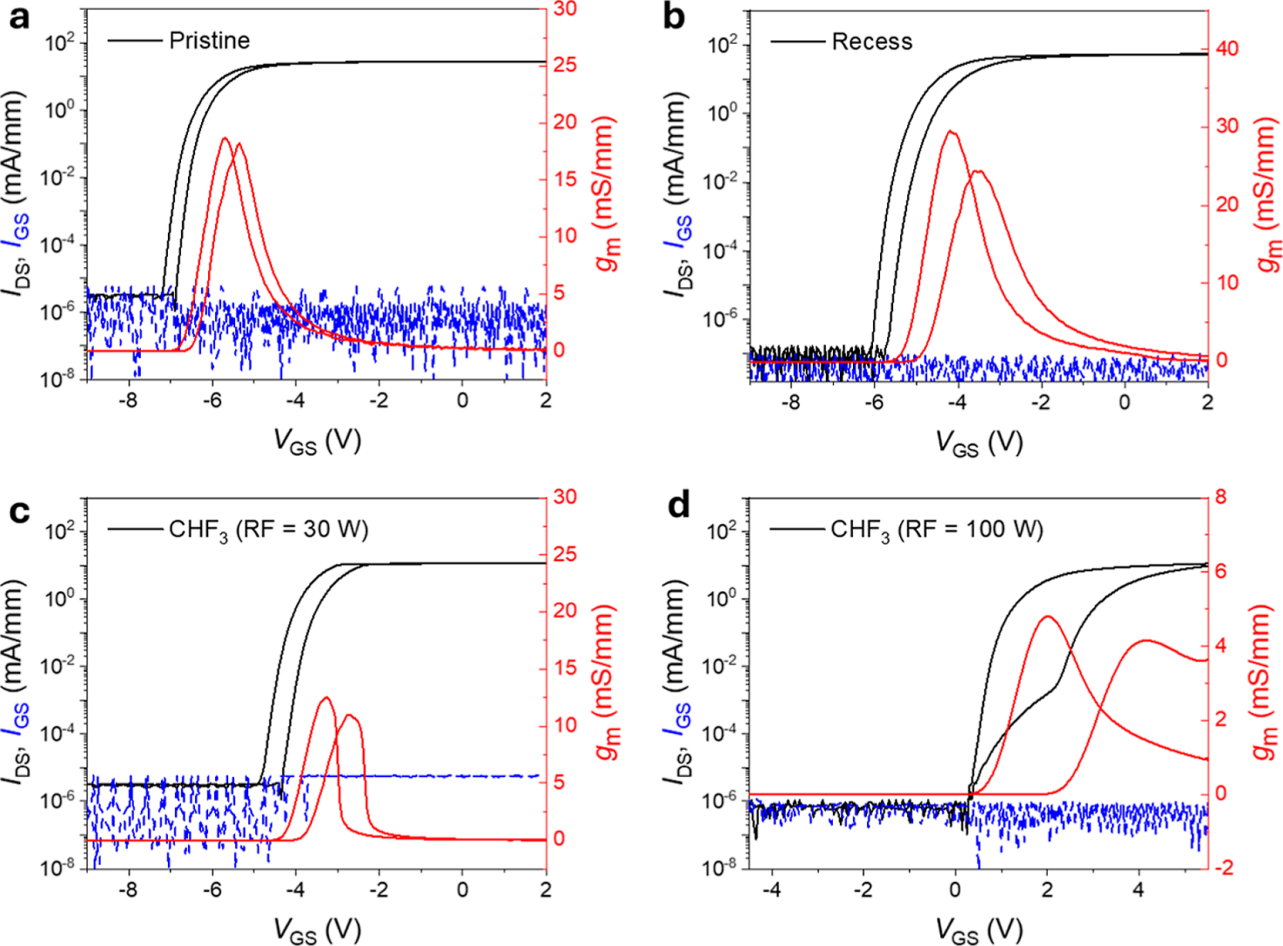
Transfer characteristics of CHF_3_-plasma-treated AlGaN/GaN HEMTs. (a–d) Transfer characteristics (*I*_DS_–*V*_GS_) and gate leakage currents of untreated (a), recessed (b), and CHF_3_-treated devices at 30 W (c) and 100 W (d) measured at *V*_DS_ = 1 V. The *V*_th_ shift from −5.70 V (untreated) to 2.00 V (CHF_3_-treated at 100 W), indicating the transition from D- to E-mode operation. The recessed device with a comparable etch depth exhibited only a moderate shift to −4.2 V, confirming the dominant role of fluorine incorporation. Gate leakage remains in the nA/mm range for both untreated and treated samples.

This progressive modulation originates from two simultaneous plasma-driven effects. First, the increased sheath potential at high RF power accelerates fluorine ions toward the surface, thereby promoting more effective barrier thinning. Second, the increased RF power raises the fluorine incorporation due to the enhanced mean energy of the ions, thus increasing the number of reactive sites in the AlGaN layer as observed in the XPS spectra.^39–41^ These mechanisms strengthen the electrostatic coupling to the channel and more effectively compensate for the polarization-induced interfacial charge. In contrast, a control device subjected solely to an equivalent recess etch without fluorination exhibits only a modest shift in *V*_th_ to −4.20 V, confirming that fluorine incorporation, rather than etching alone, governs threshold-voltage modulation. The CHF_3_ plasma treatment shows minimal effect on the off-state gate-leakage current density, which remains in the range of 0.1–1 nA/mm across all devices, indicating that junction integrity is preserved. However, as fluorine penetrates deeper to the 2DEG channel due to the partial etching of the AlGaN barrier, the negatively charged species reside closer to the channel, thereby increasing trap-mediated hysteresis and reducing the on-state current. Balancing recess depth while controlling fluorine incorporation, together with post-thermal annealing, offers a potential to suppress hysteresis and recover on-state current without compromising the intended positive *V*_th_ shift by stabilizing fluorine bonding and passivating residual plasma-induced defects.^18^

Output characteristics were measured by sweeping the drain voltage from 0 to 10 V while the gate bias was incrementally adjusted (Figure 5). For each device, gate voltage (*V*_GS_) was swept between −7 V and +5 V. The untreated HEMT exhibits a drain current density of ∼10 mA/mm at *V*_GS_ = 0 V (Figure 5a), confirming D-mode operation. The recessed control sample exhibited behavior similar to that of the pristine device, with the onset of current occurring at a slightly higher *V*_GS_ of −5 V, reflecting the reduced channel charge following removal of the GaN cap. This limited shift arises from the remaining ∼11 nm of the AlGaN barrier, which still supports substantial polarization-induced charge at the AlGaN/GaN interface.^10,42^ CHF_3_-plasma-treated devices show a systematic evolution in behavior with an increase in RF power. At an RF power of 30 W, the device remains in depletion-mode (Figure 5c), whereas treatment at 100 W induces an enhancement-mode behavior (Figure 5d), although clear saturation and well-defined pinch-off were not fully achieved. These results, consistent with prior reports on fluoride-based plasma treatments, confirm that selective CHF_3_ exposure provides a robust approach for achieving reliable E-mode operation.^18,39,43^

**Figure 5:**
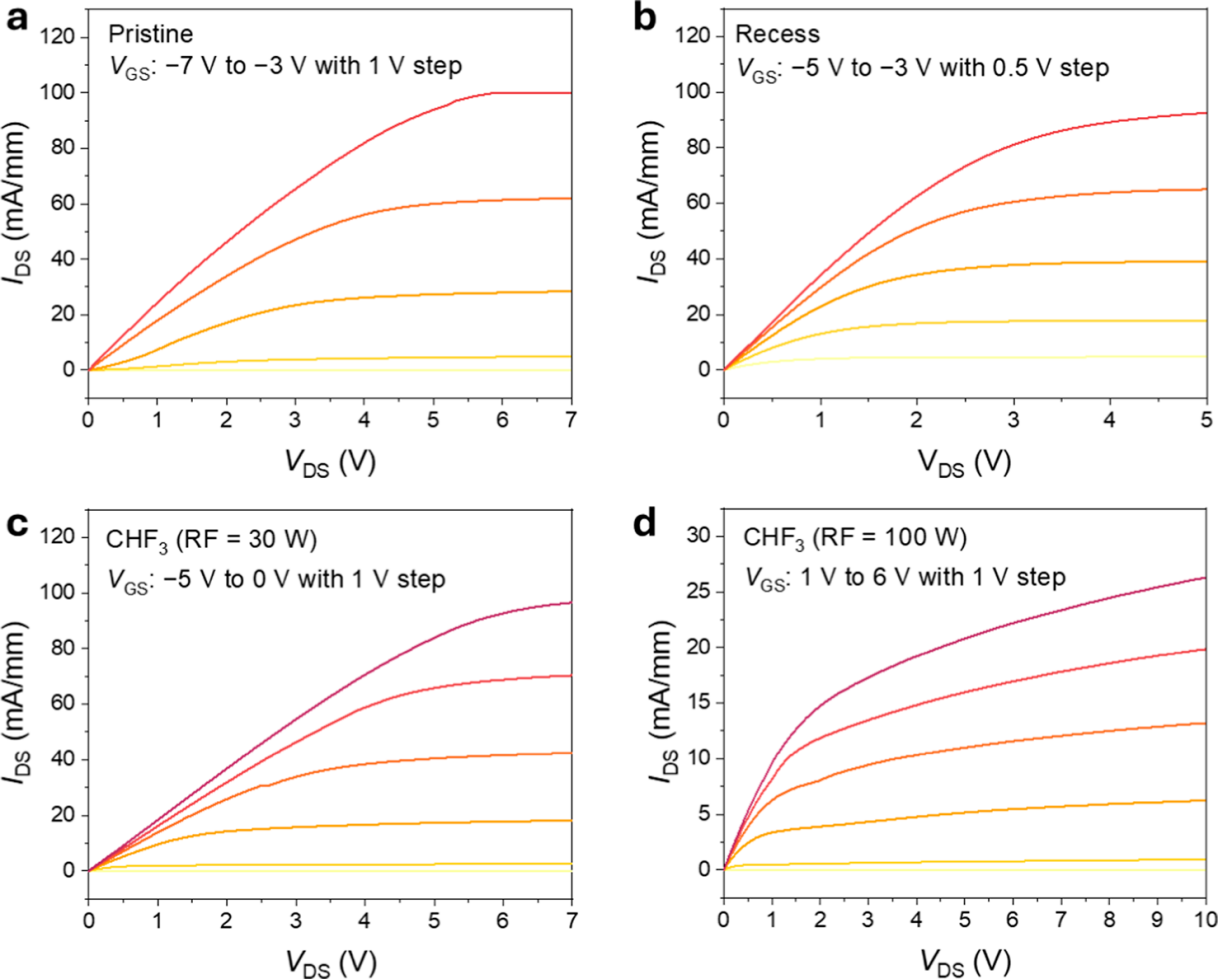
Output characteristics of CHF_3_-plasma-treated and -untreated AlGaN/GaN HEMTs. (a–d) Output characteristics (*I*_DS_–*V*_DS_) measured with *V*_GS_ swept in 0.5 or 1 V steps. The untreated device delivers substantial current even at *V*_GS_ = −3 V, confirming its depletion-mode behavior (a). The recessed device shows similar depletion-mode characteristics with a steeper *I*_DS_ response to small *V*_GS_ variations (b). CHF_3_ treatment at 30 W (c) provides partial current suppression at negative gate bias, whereas treatment at 100 W (d) leads to a pronounced suppression of drain current. These trends indicate a progressive shift toward enhancement-mode operation with increasing plasma power.

CHF_3_ plasma treatment implements a dual mechanism to modulate the threshold voltage in AlGaN/GaN HEMTs. Incorporation of an immobile and electronegative fluorine-rich layer beneath the gate partially compensates the polarization- and donor-induced positive charges and raises the local conduction-band potential while mild thinning of the AlGaN barrier enhances electrostatic coupling to the channel.^39^ Furthermore, etching induces Ga vacancies that may introduce acceptor-like states, and hydrogen species from the plasma passivate these defects, reducing trap activity and stabilizing the surface potential. However, increased fluorine penetration can introduce hysteresis via trap-mediated charging, and the reduced 2DEG density due to barrier thinning may compromise on current. Optimizing plasma parameters including bias, duration, and gas composition is therefore critical to balancing robust threshold control, device stability, and drive performance. Furthermore, postpassivation steps such as RTA under N_2_ ambient or NH_3_ plasma treatment can further enhance the device performance by additionally passivating N vacancies.^18,44^ These results establish fluorine incorporation as an effective strategy for modulating *V*_th_ without relying solely on recess etching.

## Conclusions

We demonstrate a gate-localized CHF_3_ plasma treatment that induces a self-limiting nanometer-scale recess with fluorine termination to realize E-mode AlGaN/GaN HEMTs. During plasma exposure, the incorporation of fluorine at the AlGaN surface partially neutralizes the polarization-induced positive charge. Additionally, the nanometer-scale barrier thinning enhances the electrostatic gate control and further reduces the 2DEG density at the AlGaN/GaN interface. Combining fluorine incorporation and barrier thinning shifts the *V*_th_ more positively than a recessed control device with comparable etched depth. This confirms that fluorine contributes to electrostatic modulation beyond geometric thinning of the barrier alone. Furthermore, hydrogen species introduced during treatment passivate Ga vacancies and suppress interface-trap formation, enhancing the electrical stability. Relative to conventional CF_4_-based processing, CHF_3_ treatment minimizes surface damage and suppresses trap generation, thereby enabling precise and recess-free control of *V*_th_. The fabricated devices exhibit normal off operation while maintaining acceptable gate leakage characteristics, addressing the intrinsic safety and energy-efficiency challenges associated with D-mode operation. Therefore, gate-localized fluorination via CHF_3_ plasma offers a process-compatible route to achieve E-mode GaN HEMTs without substantial modifications to existing fabrication flows, providing a promising pathway for high-power and high-frequency applications.

## Data Availability

The data related to the figures and other findings of this study are available from the corresponding author upon reasonable request.
